# Nitrogen accountancy in space agriculture

**DOI:** 10.1038/s41526-024-00428-x

**Published:** 2024-09-28

**Authors:** Kevin Yates, Aaron J. Berliner, Georgios Makrygiorgos, Farrah Kaiyom, Matthew J. McNulty, Imran Khan, Paul Kusuma, Claire Kinlaw, Diogo Miron, Charles Legg, James Wilson, Bruce Bugbee, Ali Mesbah, Adam P. Arkin, Somen Nandi, Karen A. McDonald

**Affiliations:** 1 Center for the Utilization of Biological Engineering in Space (CUBES), Berkeley, CA USA; 2 Department of Chemical Engineering, University of California Davis, Davis, CA USA; 3 Department of Bioengineering, University of California Berkeley, Berkeley, CA USA; 4 Program in Aerospace Engineering, University of California Berkeley, Berkeley, CA USA; 5 Department of Chemical and Biomolecular Engineering, University of California Berkeley, Berkeley, CA USA; 6 Department of Plant Soils and Climate, Utah State University, Logan, UT USA; 7 Zea Biosciences Inc., Walpole, MA USA

**Keywords:** Plant sciences, Aerospace engineering, Chemical engineering

## Abstract

Food production and pharmaceutical synthesis are posited as essential biotechnologies for facilitating human exploration beyond Earth. These technologies not only offer critical green space and food agency to astronauts but also promise to minimize mass and volume requirements through scalable, modular agriculture within closed-loop systems, offering an advantage over traditional bring-along strategies. Despite these benefits, the prevalent model for evaluating such systems exhibits significant limitations. It lacks comprehensive inventory and mass balance analyses for crop cultivation and life support, and fails to consider the complexities introduced by cultivating multiple crop varieties, which is crucial for enhancing food diversity and nutritional value. Here we expand space agriculture modeling to account for nitrogen dependence across an array of crops and demonstrate our model with experimental fitting of parameters. By adding nitrogen limitations, an extended model can account for potential interruptions in feedstock supply. Furthermore, sensitivity analysis was used to distill key consequential parameters that may be the focus of future experimental efforts.

## Introduction

The benefits of crop cultivation to support human spaceflight have been explored since the 1950s^1^. With the recent emphasis on longer-duration missions to the Moon and Mars (and interplanetary bases in between), human life support must maintain crew health under radically different constraints (e.g. limited availability of emergency resupply)^2–6^. The continued development of plants within the space bioprocess engineering community^7^ as a source of sustenance^8^, medicine^9^, and various other high-value life support products addresses the challenges of technology research of the National Aeronautics and Space Administration (NASA) to manage space resources and expand the human presence beyond Earth^10^.

**Fig. 1:**
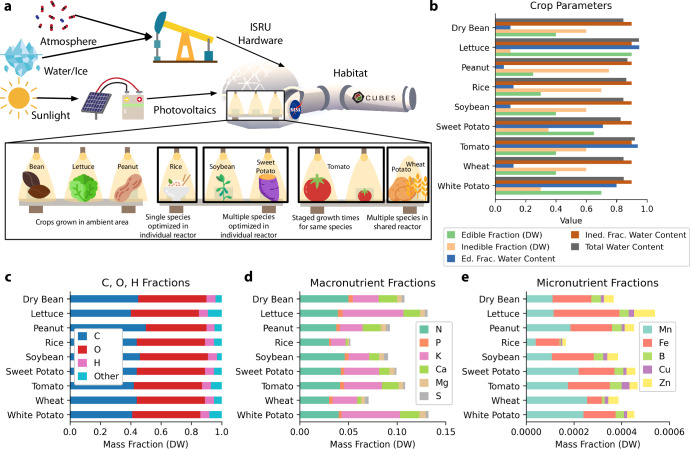
Mars-based agriculture overview. **a** Scheme for deploying agriculture systems on Mars using In Situ Resource Utilization (ISRU) with an expansion of candidate crops within habitat^11^. The expansion of crop systems includes example groupings of crops and hydroponic reactor logistics. **b** Breakdown of crop parameters for nine MEC-modeled space crops: water content fractions and edible (harvest index) and inedible fractions of biomass on a dry weight (DW) basis^11^. Values are out of 1. **c** Carbon content reference values^11^ and approximate oxygen and hydrogen fractions typical in cultivated plants^81^; “Other" fraction does not correspond numerically to (**d**, **e**) as shown here. **d** Compiled values for nitrogen and other macronutrient fractions in field-grown plants^82,83^. **e** Micronutrient fractions in field-grown plants^82,83^.

Martian agriculture (Fig. 1) has been shown to be a theoretically feasible alternative to pre-packaged meals, but caloric intake alone does not fully describe the requirements for astronaut sustainability; pharmaceutical needs must also be met^1,9,11^. Additionally, the space environment imposes a further risk of compromised food consumables and requires additional reserves of elemental carbon, nitrogen, and phosphorus^12^. Although there are some physicochemical means to recycle a subset of these elements, they are usually mass and energy intensive^13^, and they generally require additional downstream processing^14^. Given that the demand for consumable food mass scales nearly linearly with the increasing mission duration/crew size and that storage of larger quantities of food necessitates additional costs in refrigeration, storage, and power, crop cultivation provides a means for cost reduction^6,11,15^.

**Fig. 2:**
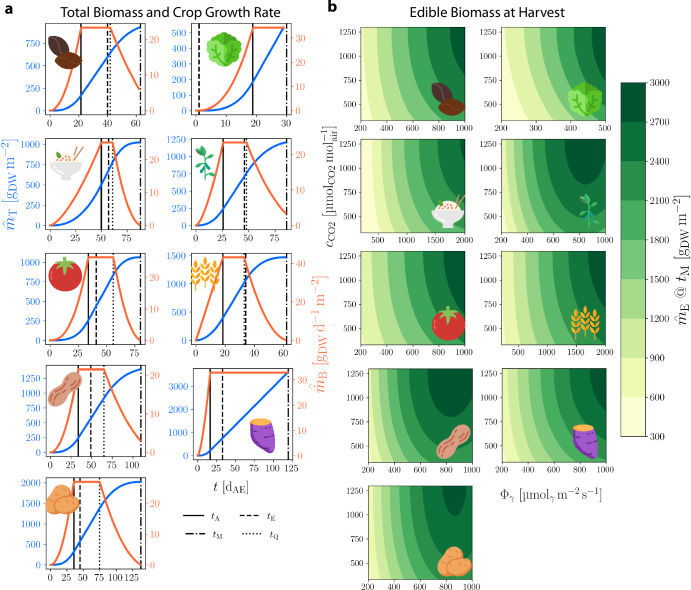
Modified energy cascade model calculations. **a** MEC model total crop biomass per area, ${\mathop{m}\limits^{\smallfrown}}_{{\rm{T}}}$ (blue), and crop growth rate per area, ${\mathop{\dot{m}}\limits^{\smallfrown}}_{B}$ (orange), for (from top left) dry bean, lettuce, rice, soybean, tomato, wheat, peanut, sweet potato, and white potato with parameters *Φ*_*γ*_ = 500 μmol_*γ*_ m^−2^ s^−1^, *c*_CO2_ = 1200 $\mu {{\rm{mol}}}_{{\rm{CO}}2}\,{{\rm{mol}}}_{{\rm{air}}}^{-1}$. Crop-specific time points [d_AE_]: *t*_A_, canopy closure; *t*_M_, harvest/maturity; *t*_E_, onset of organ formation; *t*_Q_ onset of canopy senescence. **b** MEC model contours of edible biomass accumulation, ${\mathop{m}\limits^{\smallfrown}}_{{\rm{E}}}$, for each crop terminating at *t*_M_, across *Φ*_*γ*_ and *c*_CO2_.

A primary advantage of a space bioprocess engineering^7^ is the interconnectivity and recyclability of the components of an integrated biomanufactory^16^. To date, crop cultivation has been typically been studied and characterized in many configurations (Fig. 1a) as an isolated system. There have been some exceptions from ESA’s MELiSSA (Micro-Ecological Life Support System Alternative) where crops are integrated into a biomanufactory concept and in studies on air revitalization^17–19^. Correspondingly, the established crop cultivation mathematics of NASA’s modified energy cascade (MEC) model^11,20^ are designed to model crop cultivation behavior of a single crop type in isolation, focusing on providing information relevant to traditional crop cultivation outcomes (e.g. food, environmental revitalization). This model predicts dry weight (DW) biomass production (the focus of this work) and oxygen production using photosynthetic photon flux and atmospheric carbon dioxide concentration; it is also a basis for modeling water transpiration in the crop canopy^21^. The MEC model specifies environmental conditions for a crop of interest and requires that water and nutrients are not growth limiting. The nomenclature and mathematics of the model presented here are adapted from their original forms to align more closely with engineering conventions; see the Methods section for details. In the MEC model, the change over time of the biomass per unit growing area on a DW basis in a single reactor of some crop *i*, denoted as ${\mathop{m}\limits^{\smallfrown}}_{{\rm{B}}}$ [g_DW_ m^−2^], is formulated by a differential equation as

1 $$\frac{d{\mathop{m}\limits^{\smallfrown}}_{{\rm{B}},i}}{dt}={\mathop{\dot{m}}\limits^{\smallfrown}}_{{\rm{B}},i}=\frac{{\check{m}}_{\rm{C}}}{{w}_{{\rm{C}},i}}{\mathop{\dot{n}}\limits^{\smallfrown}}_{{\rm{C}},i}$$

2 $$=0.0036\cdot \frac{{\check{m}}_{\rm{C}}}{{w}_{{\rm{C}},i}}\left({H}_{i}\cdot {\eta }_{{\rm{C}},i}\cdot {A}_{i}\cdot {\Phi }_{\gamma ,i}\cdot {Y}_{{\rm{Q}},i}\right)$$

where ${\mathop{\dot{m}}\limits^{\smallfrown}}_{{\rm{B}}}$ is areal (“per area”) crop biomass growth rate in [g_DW_ d^−1^ m^−1^], *t* is time in [d_AE_] (days after seed emergence), ${\check{m}}_{{\rm{C}}}$ is the molar mass of carbon in [${{\rm{g}}}_{{\rm{C}}}\,{{\rm{mol}}}_{{\rm{C}}}^{-1}$], *w*_C_ is the dimensionless carbon mass fraction in plant biomass [${{\rm{g}}}_{{\rm{C}}}\,{{\rm{g}}}_{{\rm{DW}}}^{-1}$], and ${\mathop{\dot{n}}\limits^{\smallfrown}}_{{\rm{C}}}$ is the daily carbon gain in [mol_C_ d^−1^ m^−2^]. The term ${\mathop{\dot{n}}\limits^{\smallfrown}}_{{\rm{C}}}$ can be represented as the product of the photoperiod *H* in [h d^−1^], the 24-hour carbon use efficiency *η*_C_ in [${{\rm{mol}}}_{{\rm{C}},{\rm{biomass}}}\,{{\rm{mol}}}_{{\rm{C}},{\rm{fixed}}}^{-1}$], the time and species dependent dimensionless fraction of photosynthetic photon flux absorbed by the plant canopy *A*, incident photosynthetic photon flux^22^ (PPF) *Φ*_*γ*_ in [μmol_*γ*_ m^−2^ s^−1^], and the quantum yield of the canopy *Y*_Q_ (a function of *Φ*_*γ*_ and atmospheric concentration of carbon dioxide, *c*_CO2_ [ppm or $\mu {{\rm{mol}}}_{{\rm{CO}}2}\,{{\rm{mol}}}_{{\rm{air}}}^{-1}$]) in [${{\rm{mol}}}_{{\rm{C}},{\rm{fixed}}}\,{{\rm{mol}}}_{\gamma \,{\rm{absorbed}}}^{-1}$]. The total areal biomass ${\mathop{m}\limits^{\smallfrown}}_{{\rm{T}}}$ in [g_DW_ m^−2^] and the edible areal biomass ${\mathop{m}\limits^{\smallfrown}}_{{\rm{E}}}$ in [g_DW_ m^−2^] for some crop *i* are calculated by

3 $${\mathop{m}\limits^{\smallfrown}}_{{\rm{T}},i}=\int_{0}^{{t}_{{\rm{M}},i}} {\mathop{\dot{m}}\limits^{\smallfrown}}_{{\rm{B}},i}\,dt$$

4 $${\mathop{m}\limits^{\smallfrown}}_{{\rm{E}},i}=f_{{\rm{E}},i}\int_{{t}_{{\rm{E}},i}}^{{t}_{{\rm{M}},i}} {\mathop{\dot{m}}\limits^{\smallfrown}}_{{\rm{B}},i}\,dt$$

where *t*_M_ is the crop-specific time of harvest or maturity in [d_AE_] and *f*_E_ is the dimensionless crop-specific fraction of daily carbon gain allocated to edible biomass after *t*_E_, which is the crop-specific time of organ formation onset in [d_AE_]. Nine crops of interest have been parameterized for use with the MEC model. For each, Fig. 2 shows the model’s biomass output (a) over time for constant *Φ*_*γ*_ and *c*_CO2_, and (b) at harvest time given varying *Φ*_*γ*_ and *c*_CO2_. A schematic view of the MEC model for *Lactuca sativa* (lettuce) is shown in Figure S1.

## Nitrogen productivity model

Nitrogen is an essential plant nutrient central to the synthesis of photosynthetic proteins and pigments. The availability of nitrogen in the rootzone is therefore a decisive factor for plant photosynthetic capacity, growth, and yield^23,24^. Modeling the effect of nitrogen on plant growth conditions becomes of paramount importance within the scope of biologically-driven mission planning since nitrogen is a limited resource that needs to be optimally allocated to ensure proper food and pharmaceuticals production and subsequent safety of the crew members^25^. A Martian mission design is a non-trivial problem, and since the decision making is partially driven by models, any uncertainty regarding their predictive capability should be taken into account. Thus, working toward a validated model that forecasts the success of crop growth given the availability of nitrogen, as well as a description of confidence in this prediction, is of great importance to this goal^26^.

A theory of nitrogen productivity (NP) was developed by Ågren in response to the complexity of other nutrient-driven plant growth models; the theory posits that plant biomass growth is determined by the total amount of nitrogen in a plant at a given time^27^. The theory assumes that for a species in fixed environmental conditions, growth can be described by the nitrogen productivity parameter, which is the quantity of biomass produced over a given time step per quantity of nitrogen in the plant, and that this value is constant during the plant’s main growth phase. NP theory is applicable when nitrogen is the limiting factor for biomass growth. The equation which describes growth has the form

5 $$\frac{d{m}_{{\rm{B}}}}{dt}={\dot{Y}}_{{\rm{N}}}\cdot {m}_{{\rm{N}}}$$

where *m*_B_ is total plant biomass on a dry weight basis in [g_DW_], *t* is time in [d], and *m*_N_ is total amount of nitrogen in the plant in [g_N_]. ${\dot{Y}}_{{\rm{N}}}$ is the nitrogen productivity, the biomass produced per day per amount of nitrogen in the plant in [${{\rm{g}}}_{{\rm{DW}}}\,{{\rm{d}}}^{-1}\,{{\rm{g}}}_{{\rm{N}}}^{-1}$]. Rearranging, nitrogen productivity is defined in quantities which can be experimentally measured:

6 $${\dot{Y}}_{{\rm{N}}}=\frac{1}{{m}_{{\rm{N}}}}\frac{d{m}_{{\rm{B}}}}{dt}$$

Ågren assumed that ${\dot{Y}}_{{\rm{N}}}$ has the form

7 $${\dot{Y}}_{{\rm{N}}}=a-{a}^{{\prime} }\frac{{m}_{{\rm{B}}}}{{m}_{{\rm{N}}}}$$

where *a* is a leading term in [${{\rm{g}}}_{{\rm{DW}}}\,{{\rm{d}}}^{-1}\,{{\rm{g}}}_{{\rm{N}}}^{-1}$] and ${a}^{{\prime} }$ is a correction term in [d^−1^]. Both are species-specific constants for fixed environmental conditions. Verkroost & Wassen suggested physically meaningful descriptions of these terms, with *a* being the product of the dimensionless efficiency of photosynthetic nitrogen (*i.e*. biologically active in photosynthesis-involved enzymes) formation from total plant nitrogen and efficiency of biomass formation from photosynthetic nitrogen [${{\rm{g}}}_{{\rm{DW}}}\,{{\rm{d}}}^{-1}\,{{\rm{g}}}_{{\rm{N}}}^{-1}$] and with ${a}^{{\prime} }$ as the degradation rate of photosynthetic nitrogen [d^−1^]^28^.

The concept of nitrogen use efficiency (NUE) was coupled with NP theory to build a framework for modeling nitrogen-limited growth which considers quantities of agricultural interest. The precise definition of NUE has varied depending upon its intended application, but in general it refers to a biomass yield given a quantity of exogenous nitrogen provided per plant, or per area for field grown crops. Here we introduce three model quantities derived from a general approach to NUE calculations^29,30^. The nitrogen productivity is given the form

8 $${\dot{Y}}_{{\rm{N}}}={\eta }_{{\rm{N}}}\cdot {\mu }_{{\rm{N}}}\cdot {\eta }_{{\rm{u}}}$$

The NUE quantity, *η*_N_ [${{\rm{g}}}_{{\rm{DW}}}\,{{\rm{g}}}_{{\rm{N}}}^{-1}$], is the biomass (dry weight basis) produced per average total mass of N in the plant over a time period. The second quantity, relative nitrogen accumulation rate (RNAR), *μ*_N_ [d^−1^], is the mean relative accumulation rate of N over a time period. The third, uptake efficiency, *η*_u_ [dimensionless or (g d^−1^)/(g d^−1^)], is the ratio of a plant’s current uptake rate of nitrogen to its maximum. It is intended to account for abiotic effects on uptake such as temperature or pH of nutrient support solution^31,32^. To our knowledge, no modeled data for *η*_u_ are reported in literature; in this work we suppose that it is equal to 1. Its presence provides a path forward within the space agriculture community for directed research. A simple assumption is to calculate values of *η*_N_ and *μ*_N_ from experimental measurements by

9 $${\eta }_{{\rm{N}}}=\frac{{m}_{{\rm{B}}}({t}_{2})-{m}_{{\rm{B}}}({t}_{1})}{{\bar{m}}_{{\rm{N}}}({t}_{2},{t}_{1})}$$

10 $${\mu }_{{\rm{N}}}=\frac{\ln {m}_{{\rm{N}}}({t}_{2})-\ln {m}_{{\rm{N}}}({t}_{1})}{({t}_{2}-{t}_{1})}$$

where *t*_2_ and *t*_1_ are the time points at the end and beginning of a time step, typically^33^ in [d] or [week], and ${\bar{m}}_{{\rm{N}}}$ is the mean total N in the plant over a time step (*t*_2_ − *t*_1_) in [g_N_]. The appropriate time step for both equations depends on harvest interval, the particular crop, and its growth stage. Finally, given the model output curves shown in Fig. 2, we assume that the total mass of N in the plant, *m*_N_, throughout its life cycle can be described by the three-parameter logistic function, giving

11 $${m}_{{\rm{N}}}(t)=\alpha \frac{{m}_{{\rm{N}}0}\cdot K\cdot {e}^{rt}}{(K-{m}_{{\rm{N}}0})+{m}_{{\rm{N}}0}\cdot {e}^{rt}}$$

and finally

12 $$\frac{d{m}_{{\rm{B}}}}{dt}={\eta }_{{\rm{N}}}\cdot {\mu }_{{\rm{N}}}\cdot {\eta }_{{\rm{u}}}\cdot {m}_{{\rm{N}}}$$

where *m*_N0_ is the initial total mass of nitrogen in the plant in [g_N_], *K* is the limiting value of *m*_N_ in [g_N_], *r* is the governing rate in [d^−1^], *t* is time in [d], and *α* is a dimensionless scaling factor.

While our study on nitrogen dynamics in hydroponically grown lettuce has broad applicability to general agriculture, it is particularly relevant to space agriculture due to the unique constraints of long-duration space missions. In space, resource optimization, closed-loop nutrient management, and minimization of physiological stress are critical for maintaining crew health and mission sustainability. The development of efficient nitrogen use strategies and the potential utilization of in situ resources, such as amino acids and peptides found on other astronomical bodies^34,35^, are essential for supporting bioregenerative life support systems in space. This contributes to both terrestrial agricultural advancements and the specialized needs of space agriculture, addressing the challenges of sustainable food production in extraterrestrial environments.

## Model integration

**Fig. 3:**
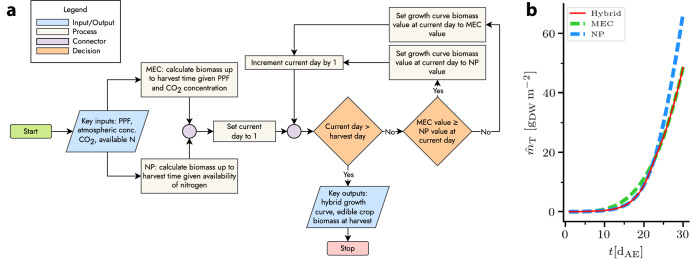
Hybrid MEC-NP modeling. **a** Algorithm by which the growth curves of the MEC and NP models act as limiting factors in a hybrid growth curve. **b** Example of hybrid growth curve, limited first by nitrogen, then by photosynthetic photon flux, atmospheric CO_2_ concentration, or both.

The simplest strategy for the use of both NP and MEC models is to use the MEC model by default and a NP model in the case of nitrogen-limited growth. A slightly more complex option is to combine each model’s growth curves such that either can act as a limiting factor. This approach accounts for changing limitations during the growth period. A flowchart representation of a basic algorithm is shown in Fig. 3 alongside example output.

A fully integrated MEC-NP model should also include limiting effects of off-nominal growth environment parameters such as air temperature, relative humidity, and air circulation on biomass generation. Additionally, in hydroponic and aeroponic systems, temperature, pH, dissolved oxygen, and molecular concentrations in the nutrient support solution can affect biomass growth^36,37^. It is important to consider that both traditional selection and breeding as well as genetic engineering have produced crop plants that can adapt to a range of abiotic stresses^38^; development of specialized crop lines would be expected for use in spaceflight^39^ and certain limiting effects could be minimized. For comparison with MEC model, a NP model must account for required growing area and edible fraction of biomass, which in nitrogen-limited growth conditions may be different than the MEC reference values.

## Results

An experiment was designed to measure the nitrogen content and biomass accumulation over time in hydroponically grown *Lactuca sativa* cv. “Waldmann’s Dark Green”, a looseleaf lettuce reference cultivar (Fig. S2), in deficient, normal, and excess nitrogen conditions. Lettuce has served as a useful model for space agriculture^11,40,41^. A batch strategy was used for the hydroponic systems’ nutrient support solution (NSS); no nitrogen was added to the NSS reservoirs after the initial dose. The initial baseline concentration of nitrate (${{\rm{NO}}}_{3}^{-}$) was equal in all N conditions, while the initial ammonium (${\rm{NH}_{4}}^{+}$) concentration was varied (see Tables S2 and S3). The nitrogen conditions were as follows: deficient (1.0 mM nitrate, 1.5 mM ammonium); normal (1.0 mM nitrate, 6.5 mM ammonium); excess (1.0 mM nitrate, 11.5 mM ammonium). The methodology and growth environment are detailed in the Methods section.

**Fig. 4:**
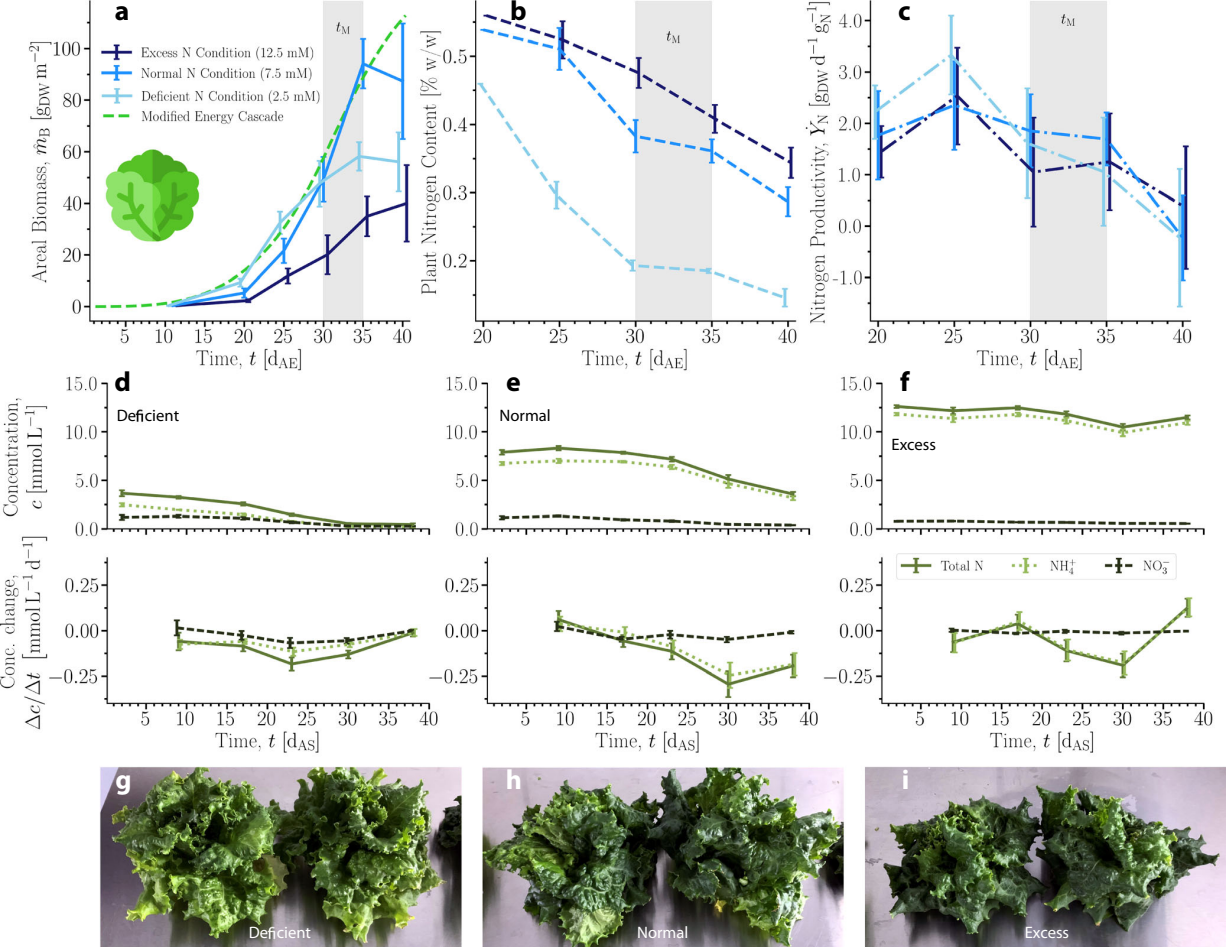
Nitrogen Productivity Studies. **a** Measured areal biomass and MEC model curve calculated with *Φ*_*γ*_ = 225 μmol_*γ*_ m^−2^ s^−1^ and *c*_CO2_ = 525 ppm. **b** Measured fresh weight percentage of total nitrogen in plants over time. A single measurement was performed for each condition at 20 d_AE_. **c** Nitrogen productivity calculated from measured biomass and nitrogen content. Error bars represent propagated error. **a**–**c** Time range highlighted in gray is specified harvest time, *t*_M_, plus 5 d^11^. Error bars represent one SD. For each data point, 5 ≤ *N* ≤ 10. **d**–**f** Concentration and change in concentration of nitrogen measured in the NSS over time by experimental condition (deficient, normal, excess). Error bars represent 1 SD. *N* = 3 for each data point. **g**–**i** Photos of lettuce crops during main growth phase at 35 d_AE_ grown in deficient, normal, and excess nitrogen conditions, respectively.

The seed germination percentages were 71%, 81%, and 62% for deficient, normal, and excess N levels, respectively. The particular hydroponic system used shows an average germination percentage of 85% for lettuce given normal N. The biomass data were compared to the output of the MEC model (Eq. (2)) with inputs of the average values of *Φ*_*γ*_ and *c*_CO2_ observed in the hydroponic system (Fig. 4a); see Fig. S6a, b for the growth rate [g_DW_ d^−1^] and relative growth rate^42^ [${{\rm{g}}}_{{\rm{DW}}}\,{{\rm{d}}}^{-1}\,{{\rm{g}}}_{{\rm{DW}}}^{-1}$].

The excess nitrogen condition resulted in the lowest average biomass at harvest time (defined as 30–35 d_AE_), while the normal condition resulted in the highest. The MEC prediction of biomass was most similar to the growth curve of the normal nitrogen condition over the time period shown. Its predicted values were similar to the deficient condition measurements until 30 d_AE_, and its predictions were consistently higher than the measured biomass in the excess condition. This demonstrates the need for extension of the MEC model to account for nitrogen availability, whether in deficit or excess.

For all conditions, the average fresh weight percentage of N decreased over time (Fig. 4b). Toward *t*_M_, the average mass fraction of plant N in the normal condition was smaller than that of the excess condition, yet the plants in the normal condition achieved greater average biomass, indicating that excess ammonium may have induced growth-inhibiting stresses. In the deficient condition, the average plant N content was lower than in the other conditions, but the average biomass was higher than the plants in the excess condition and was similar to that of the plants in the normal condition up to 30 d_AE_. This indicates that the plants in deficient conditions may have used N for growth more efficiently than plants in the other conditions at the expense of their average biomass at *t*_M_. The average nitrogen content across all conditions over time on a fresh weight basis was 0.36 ± 0.13%, similar to a reported value of 0.16% for iceberg lettuce measured by the same analytical method^43^. In terms of non-normalized mass of N (Fig. S6c), the plants in normal and excess conditions contained an increasing average mass of nitrogen through the harvest period, while the plants in the deficient condition initially increased before leveling off around 25 d_AE_.

Nitrogen productivity (${\dot{Y}}_{{\rm{N}}}$) was calculated from measured biomass and nitrogen content data (Fig. 4c). The overall average nitrogen productivity under the normal N condition was $1.33\pm 0.79\,{{\rm{g}}}_{{\rm{DW}}}\,{{\rm{d}}}^{-1}\,{{\rm{g}}}_{{\rm{N}}}^{-1}$; under the deficient and excess conditions, it was 1.49 ± 0.99 and $1.90\pm 1.32\,{{\rm{g}}}_{{\rm{DW}}}\,{{\rm{d}}}^{-1}\,{{\rm{g}}}_{{\rm{N}}}^{-1}$, respectively. The average of ${\dot{Y}}_{{\rm{N}}}$ across all conditions was $1.47\,{\pm}\,0.99\,{{\rm{g}}}_{{\rm{DW}}}\,{{\rm{d}}}^{-1}\,{{\rm{g}}}_{{\rm{N}}}^{-1}$. Empirical values in literature for ${\dot{Y}}_{{\rm{N}}}$ in other plants are on the order of 10^−1^ to 10^0^, though it should be noted that these mostly describe woody plants^27^. The value of nitrogen productivity appears to decrease near the end of the measured period, indicating that the assumptions of the NP model may have limitations outside of the main growth phase.

The molar concentrations of nitrate and ammonium in the NSS reservoirs, *c* [mM], were measured from 2–40 d after sowing (d_AS_, preceding d_AE_ by 3 d). The concentrations and changes in concentrations over time are shown in Fig. 4d, e, f for each N condition. The concentrations over time grouped by molecular form of N are shown in Fig. S7, and the relative changes of the concentrations from the initial values are shown in Fig. S8. In the deficient N condition, the rate at which both forms of N were depleted from the NSS did not show any large fluctuations over the course of the experiment. In the normal and excess N conditions, nitrate was depleted at a mostly constant rate over the time course, but ammonium depletion accelerated. The measured increase in ammonium concentration at 38 d_AS_ in the excess N condition occurred around the time that biomass was leveling off. This is possibly due to the plants beginning to bolt, at which time they reallocate resources to to transition from vegetative growth to flowering, as a response to the stress in this condition.

By 30 d_AS_, the plants in the deficient condition had depleted almost all available nitrogen but were still able to produce biomass; vascular plants can effectively take up nitrogen even when concentration is relatively low^44^. In all conditions, the depletion of nitrogen continued even as a number of plants were harvested and removed from the system every 5 days (Fig. S9). Representative photos of plants from each condition at the end of the harvest period are shown in Fig. 4g–i.

**Fig. 5:**
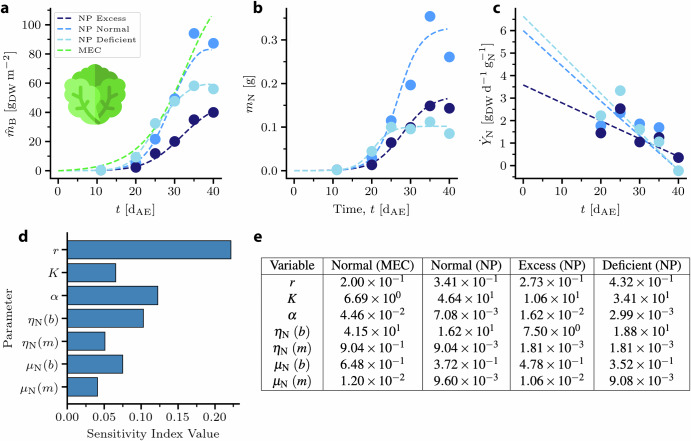
Lettuce model comparison. **a** Experimental data (circles) and NP model fits (lines) for lettuce biomass predicted by the MEC model and grown in 3 different nitrogen conditions. **b** Fitting of *m*_N_ by values of *r*, *K*, and *α* to experimental mass of N in plant. **c** Fitting of ${\dot{Y}}_{{\rm{N}}}$ by functions for *η*_N_ and *μ*_N_ to nitrogen productivity calculated from experimental data. **d** Sensitivity analysis result based on the ranges defined by the fitting procedure. The *y* − axis denotes the variable of interest wile the *x* − axis represents the corresponding variable’s index value. **e** Fitted NP parameter values for MEC baseline and experimental N conditions. *r*: governing rate in [d^−1^]; *K*: limiting value of *m*_N_ in [g_N_]; *α*: dimensionless scaling factor; *η*_N_: nitrogen use efficiency in [${{\rm{g}}}_{{\rm{DW}}}\,{{\rm{g}}}_{{\rm{N}}}^{-1}$]; *μ*_N_ relative nitrogen accumulation rate in [d^−1^].

The parameters of the NP model (Eqs. (11) and (12)) were fitted to the MEC curve as a baseline as well as to the experimental curves (Fig. 5a). The NP parameter values fitted to the MEC growth curve were not constrained by the experimental data. The fits to the experimental curves were constrained by the measured values of *m*_N_ and measurement-derived calculations of ${\dot{Y}}_{{\rm{N}}}$ over time (Fig. 5b, c); the linear approximations of ${\dot{Y}}_{{\rm{N}}}$ may not be representative of its behavior prior to the time period of the data. It was assumed that *α* and *K* did not vary with time since they represent scaling and limiting factors, respectively. The governing rate of N accumulation in the plant, *r*, was also assumed to be time-independent. The initial total mass of nitrogen available, *m*_N0_, was assumed to be on the order of 10^0^ mg. RNAR (*μ*_N_) and NUE (*η*_N_) were approximated as varying linearly with time and were fitted by *y* − intercept (*b*) and slope (*m*). The fitted parameters are shown in Fig. 5e. Planting density and fresh weight water content were used as described above to compare the NP curves to the MEC curve.

Due to the inclusion of various mechanistic equations to capture the kinetics of growth, the model was potentially overparameterized, *i.e*. more parameters existed than were necessary to fit the available data. To address this issue and to identify the most important parameters, a sensitivity analysis was performed to help determine which parameters had the greatest impact on the model predictions (Fig. 5d). Sensitivity analysis is closely related to uncertainty quantification (UQ) and is a standard tool to asses the physical impact of the parameterization on the model predictions^45^.

## Model analysis

This section describes the analysis of the model’s predictive capability to capture the fundamental effects of nitrogen content on growth. First, ranges and estimates regarding the model parameters were acquired. As explained in the Results section, the model parameters were fitted to several experimental variables. It was assumed that the parameters were condition-dependent. Thus, a different set of parameters was estimated for each N condition. Overall, the model adequately captured the experimental *m*_B_ and *m*_N_ points, while it was able to reproduce the decreasing trend in the ${\dot{Y}}_{{\rm{N}}}$ data.

A global sensitivity analysis (GSA) was performed to quantify the effect of the model parameters on the model predictions^46^. The quantity of interest (QoI) in this case was the area under the curve of the biomass concentration over time, a representative variable for the entire dynamic behavior of the system. It was assumed that each variable followed a uniform distribution. The lower and upper bounds for each distribution were determined by examining Fig. 5e. For each parameter, represented as a row in the table, the minimum and maximum values across the columns were identified and used as lower and upper bounds respectively. The results shown in Fig. 5d illustrate the relative impact of the model variability on the dynamic behavior of the system. It was observed that the growth evolution is affected strongly by approximately half of the of variables. In GSA, it is common to observe an uneven impact of parameters on the variability of the QoI^47^, while this phenomenon is directly tied with the physical meaning of each parameter. Here, the most influential parameter seemed to be the rate of N accumulation, *r*, which directly ties to NP theory. However, while *r* was highest in the deficient condition, the resulting biomass was lower than in plants under the normal condition, suggesting that the effects of off-nominal N reduced total biomass accumulation, and are accounted for by *K*.

**Fig. 6:**
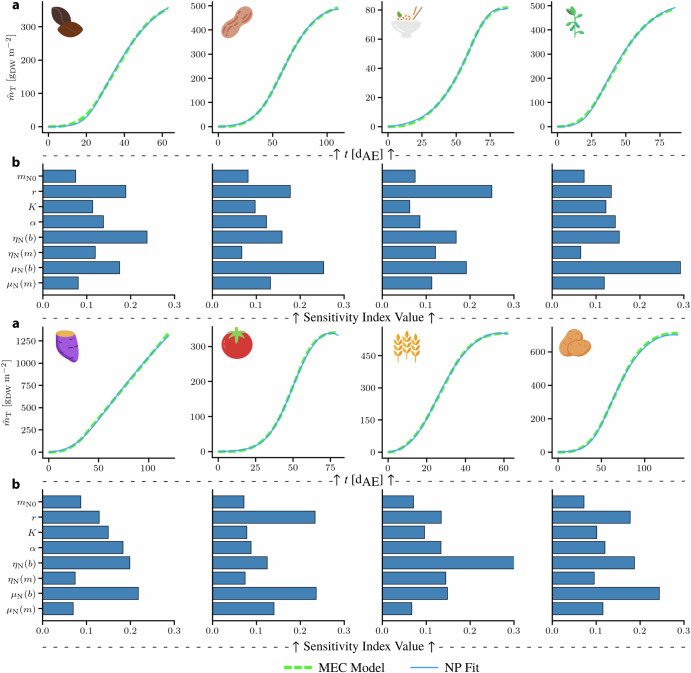
MEC and NP Model Projection Comparison. **a** NP model fits to MEC growth curves (*Φ*_*γ*_ = 225 mol_*γ*_ m^−2^ s^−1^, *c*_CO2_ = 525 ppm) for dry bean, peanut, rice, soybean, sweet potato, tomato, wheat, white potato. **b** Sensitivity analysis result based on the ranges defined by the fitting procedure. The *y* − axis denotes the variable of interest wile the *x* − axis represents the corresponding variable’s index value.

Subsequently, the dynamic behavior and sensitivity of other crops to the NP model parameters were analyzed similarly to lettuce. Though experimental data for the other crops were not available, some appropriate parameter values for them were obtained by fitting the NP model to the predictions of the MEC model. Once the parameters were fitted, it was assumed that they followed a uniform distribution by perturbing them by ± 10% around their nominal values. This is a common approach when a prior distribution for parametric uncertainty is not well established^45^. From the results shown in Fig. 6, it is evident that the NP model was able to perfectly fit the MEC model predictions. Similar to the results from lettuce growth, the rate of N accumulation, *r*, still appears to play a significant role in the growth dynamics of most plants, being one of the most influential parameters in most crops. Collectively, other parameters that stand out as affecting growth are *η*_N_ and *μ*_N_. A similar trend was observed in lettuce; however, the effect of *r* in lettuce was much more pronounced. A key general observation is that when nitrogen dynamics are included in the prediction of growth dynamics, the variation of the corresponding parameters of N evolution over time seems to induce most of the variation in growth, exemplifying the effect of N. It should be noted that the validity of the GSA for the crops, in the absence of experimental data, is limited to this particular model structure and distribution of its parameters. The GSA indices reveal the relative significance of the parameters included in the model and do not take into consideration other factors that might affect crop growth unless they are explicitly accounted for in the model. In addition, since the GSA relies on sampling techniques from the assumed distribution (uniform here), a shift in distribution can have an impact on the sensitivity indices. Nevertheless, the same analysis can be applied to more comprehensive models that describe more complex growth dynamics. It is important to recognize that crop growth is influenced by a multitude of factors, including environmental conditions and other nutrient availability. Future work should aim to integrate these additional factors into the models to provide a more holistic understanding of crop growth dynamics.

## Discussion

Plant growth and development depends largely on the available concentration of nitrogen in growth conditions, whether in soil or in soil-less hydroponic systems. The mineral nutrients are absorbed by plant roots and therefore their availability in the soil or hydroponic medium is critical for its absorption to maintain normal physiological processes^48^. While the batch strategy for nutrient provision and the environmental growing conditions provided baseline data for testing the NP model, behavior under conditions expected in a life support environment could be explored by increasing the CO_2_ concentration to around 1200 ppm, maintaining constant nutrient concentrations in the NSS, and periodically replacing the NSS as part of a semi-continuous process^26,49,50^.

Nitrogen plays an essential role in the structure of amino acids and N-bases; therefore its depletion in the growth medium may halt important physiological processes crucial for plant growth^51^. In general, a plant in N stress conditions exhibits symptoms such as stunted growth, yellowing of the leaves, leaf death, and reduction in chlorophyll production, and therefore ultimately contributes heavily to the reduction of overall crop yield^52^. In lettuce, nitrogen deficiency stress conditions result in a slower growth rate and reduction in water content^53^. Given the similar responses among diverse crop plants to nitrogen deficiency^54^, the reduction in lettuce biomass would be expected. Alternatively, an excess of N availability can also negatively affect plant growth parameters such as root and shoot biomass^48^. It is expected that the experimental growth curves, and thus the NP fitting parameters, depend on the form of N that is provided in deficient, normal, and excess concentrations. This is due not only to how the N is taken up and used, but also to its effects on the NSS. Relevant to the experiment described herein, elevated ${{{\rm{NH}}}_{4}}^{+}$ concentration relative to ${{{\rm{NO}}}_{3}}^{-}$ reduces the pH of the NSS and, when taken up by the plant, can inhibit the uptake of other cations^49^, demonstrating the importance of the choice of nutrient feed strategy and of monitoring and control systems. Figure S5 shows the pH measurements during the course of the experiment; notably, of those which were out of range, the greatest quantity and magnitude were in the normal N condition, yet its average biomass was greatest, indicating that the detrimental effects on biomass observed in the excess nitrogen condition may be influenced by additional factors^55^.

As an alternative to conventional soil production, growing lettuce hydroponically is a popular approach, especially in urban settings, uncultivated lands, and other constrained environments. Lettuce plants grown in solid substrate and hydroponically within the same environment showed no significant differences in or shoot weight or morphological features (except enhanced root growth in hydroponics)^56,57^, but biomass yield is only one metric of many used to evaluate a crop production component of a life support system. For example, the nutritional profile must be considered as well, as differences in the concentrations of starches, sugars, and other bioactive compounds have been observed depending on the growth system as well as the amount and molecular form of N provided^56,58,59^.

The observations made in our study, conducted in a water-based hydroponic system, may not be directly applicable to soil- or growing media-based systems. Soil or non-inert growing media can significantly impact nitrogen dynamics in the root zone due to factors such as the microbiome composition and its activity, organic matter decomposition, and interactions with soil particles. These factors can alter nitrogen availability and uptake efficiency compared to the relatively controlled environment of hydroponic systems. Future studies should consider these differences and investigate nitrogen dynamics in various growing media to better understand their implications for space agriculture and other applications.

The trade-off between using traditional fertilizers and In Situ Resource Utilization (ISRU) for plant growth becomes apparent in such missions. While fertilizers are a proven method for providing nutrients, their use in space is limited due to the constraints of weight, volume, and stability. ISRU, on the other hand, offers the potential to harness available resources for plant growth. However, the implementation of ISRU technologies is still in its nascent stage and requires further research and development. Organic nitrogen sources, such as amino acids and peptides, are promising alternatives for nitrogen supply in space agriculture as they are prevalent in biological wastes via loop-closure. These compounds can be collected using advanced extraction and processing technologies, potentially involving bio-mining with engineered microbes. Other nitrogen sources, such as nitrates and ammonia, can be generated through ISRU technologies. Plants can efficiently take up amino acids and peptides through their roots, offering a viable pathway to enhance nitrogen use efficiency and reduce reliance on traditional inorganic fertilizers in space agriculture^60,61^.

Our study focuses on nitrogen limitations due to their critical role in plant growth and its availability in the Martian atmosphere, making it a key factor for space agriculture. However, other nutrients, such as phosphorus, which is present in a locked P_2_O_5_ state on Mars^11^, could also become limiting. Our modeling approach, based on nitrogen dynamics, can be adapted to study other nutrient limitations by adjusting parameters to account for specific nutrient dynamics. This adaptability makes our approach transferable and valuable for comprehensive nutrient management in space agriculture, ensuring the resilience of agricultural systems for long-duration space missions.

As for the next steps, the developed model provides a robust framework that can be extended to other crops. Each crop will have its specific nitrogen requirements, growth patterns, and responses to various nitrogen conditions. Comparative studies among different crops can shed light on their suitability for cultivation in a space environment, considering their nutrient requirements, growth rate, and yield. Comparing the findings in this work to recent MEC publications, the model aligns well with the ongoing research trends. The focus on resource efficiency, especially the efficient use of nutrients, is a common theme. However, this work extends it by specifically modeling the impact of different nitrogen conditions on plant growth, providing a tool for more nuanced nutrient management.

It is important to underline the appropriate use of the model and understand its limitations. While the model provides valuable insights into plant growth under different nitrogen conditions, it is based on the assumptions made and the data used for calibration. It may not capture the full complexity of real-world plant growth, particularly under extreme or unforeseen conditions. Therefore, the model should be used as an approach and as a tool for guidance, not as an absolute predictor. In terms of biological relevancy, the parameters used in the model are grounded in known biological processes. They represent various aspects of plant growth, such as nitrogen uptake, metabolic rate, and growth responses to different nitrogen conditions. However, the exact mechanisms and how they are influenced by various factors can be complex and might not be entirely captured by the model. Exploration of growth models^62^ to develop a more generalized mathematical description of nitrogen-limited biomass generation will be beneficial. Assigning physiological meaning to any parameters as well as extending the model to capture responses to external conditions will be important milestones in development of a deterministic model.

Modeling a time-varying environment is an important aspect of accurately modeling plant growth. Environmental factors such as light intensity, temperature, and carbon dioxide levels can fluctuate over time and affect plant growth. Future iterations of the model could incorporate these dynamic elements to provide a more accurate representation of plant growth under varying conditions. Development of an optimal growing strategy may include intentional changes as well; for example, dynamic planting density^11^ and N concentration in the NSS based on the crop’s growth stage could increase efficiency or yield. Finally, the model could be further refined by considering different stages of the plant life cycle, as mentioned in the work of Weih^29^. Plants exhibit different nutrient requirements and growth patterns during various stages of their life cycle (e.g., vegetative growth, flowering, and fruiting). *L. sativa* is the simplest case among the crops of interest as only vegetative growth is important for production of edible biomass, but in other crops, partitioning and accumulation of nitrogen in different organs also exhibit dynamic behavior by growth stage and by nitrogen availability, so the presence and age of a given organ may influence total nitrogen content of the plant^63,64^. Measuring the response of organ formation and growth in terms of NP and incorporating the growth stages into the model could provide a more detailed and accurate understanding of plant growth under different nitrogen conditions.

The experimental methods used to package and process biomass and extract nitrogen for measurement will lead to differing results^65^. Plant tissue showed varying nitrogen content depending on whether it was dried by heat, vacuum, or freezing prior to assaying^66^ as well as by the assay chosen^66,67^. Non-destructive assaying methods such as near infrared spectroscopy could facilitate frequent and localized measurements of nitrogen in the plants’ aerial portions^68^. Standardizing the analytical methodology will improve the utility of the model.

## Methods

### Nomenclature

Nomenclature reformation considered chemical engineering conventions, IUPAC^69^ and IUPAP^70^ documentation, and intuitive understanding. Variables and subscripts which refer to quantities are typeset in italic, while descriptive subscripts are in roman. Diacritics above variables are used to denote per time ($\dot{\square }$) and per area ($\mathop{\square }\limits^{\smallfrown}$). Mnemonically, one might think of the inverted breve above areal variables as resembling a surface; this was derived from the idea that a normal breve, ($\breve{\square }$), might signify volumetric variables as a vessel to be filled.

**Table:** 

MEC nomenclature reformation			
Variable	Description	Unit	Former Variable^11^
*a*	Empirical exponent	–	n
*c*_CO2_	Concentration of CO_2_, molar	$\mu {{\rm{mol}}}_{{\rm{CO}}2}\,{{\rm{mol}}}_{{\rm{air}}}^{-1}$	[CO_2_]
*f*_E_	Fraction of edible biomass after *t*_E_	–	XFRT
*g*_atm_	Atmospheric aerodynamic conductance	mol_water_ s^−1^ m^−2^	g_A_
*g*_sfc_	Canopy surface conductance	mol_water_ s^−1^ m^−2^	g_C_
*g*_sto_	Canopy stomatal conductance	mol_water_ s^−1^ m^−2^	g_S_
*h*_R_	Relative humidity	–	RH
${\mathop{\dot{m}}\limits^{\smallfrown}}_{\rm{B}}$	Biomass per time, areal	g d^−1^ m^−2^	CGR
${\mathop{m}\limits^{\smallfrown}}_{{\rm{E}}}$	Biomass, areal, edible	g m^−2^	TEB
${\mathop{m}\limits^{\smallfrown}}_{{\rm{T}}}$	Biomass, areal, total	g m^−2^	TCB
$\check{m}$	Mass, molar	g mol^−1^	MW
$\mathop{\dot{n}}\limits^{\smallfrown}$	Moles per time, areal	mol d^−1^ m^−2^	DCG, DOP
${\mathop{\dot{n}}\limits^{\smallfrown}}_{{\rm{ps}},{\rm{gross}}}$	Gross canopy photosynthesis	*μ*mol_C_ s^−1^ m^−2^	P_GROSS_
${\mathop{\dot{n}}\limits^{\smallfrown}}_{{\rm{ps}},{\rm{net}}}$	Net canopy photosynthesis	*μ*mol_C_ s^−1^ m^−2^	P_NET_
*P*_atm_	Total atmospheric pressure	kPa	P_ATM_
${p}_{{\rm{S}}}^{\star }$	Saturated vapor pressure	kPa	VP_SAT_
*T*_D_	Temperature, dark cycle	^∘^C	T_DARK_
*T*_L_	Temperature, light cycle	^∘^C	T_LIGHT_
*t*_sol_	Length of local sol	h d^−1^	D_PG_
${\mathop{\dot{V}}\limits^{\smallfrown}}_{{\rm{trs}}}$	Daily transpiration rate	L d^−1^ m^−2^	DTR
*w*_C_	Biomass carbon fraction	–	BCF
*Y*_O2_	Oxygen production factor	${{\rm{mol}}}_{{\rm{O}}2}\,{{\rm{mol}}}_{{\rm{C}}}^{-1}$	OPF
*Y*_Q_	Canopy quantum yield	${{\rm{mol}}}_{{\rm{C}},{\rm{fixed}}}\,{{\rm{mol}}}_{\gamma ,{\rm{absorbed}}}^{-1}$	CQY
*Δp*^⋆^	Vapor pressure deficit	kPa	VPD
*η*_C_	Carbon use efficiency, 24 hr	${{\rm{mol}}}_{{\rm{C}},{\rm{biomass}}}\,{{\rm{mol}}}_{{\rm{C}},{\rm{fixed}}}^{-1}$	CUE_24_
*σ*_*N*_	Density, areal, numeric	m^−2^	–
*Φ*_*γ*_	Photosynthetic photon flux	*μ*mol_*γ*_ m^−2^ s^−1^	PPF
*Φ*_*γ*,E_	Photosynthetic photon flux, effective	*μ*mol_*γ*_ m^−2^ s^−1^	PPF_E_

### Lettuce cultivation

The lettuce plants were grown in a hydroponic rack system within a clean room operated by Zea Biosciences in which air temperature, relative humidity, and atmospheric concentration of CO_2_ were controlled. The grow rack consisted of 3 independent shelves, each with a NSS reservoir. The pH, water temperature, total dissolved solids (TDS), dissolved oxygen (DO), nitrate concentration, and ammonium concentration in the reservoirs were monitored. The following instruments were used to perform measurements: Hanna HI98103 (pH), Hanna HI98129 (pH, TDS, temperature), Milwaukee Instruments MW600 (DO), and Horiba LAQUAtwin NO3-11 (nitrate). Ammonium concentration was determined by a spectrophotometric method adapted from Kempers & Kok^71^. The photosynthetic photon flux was set by adjusting the height of the shelves. A Hydrofarm LGBQM2 PAR meter was used to measure photosynthetic photon flux until the 4th week of growth, at which time the size of the plants was too large for measurements to be taken.

Controlled environmental set points and the measured ± 1 standard deviation over the course of the experiment were as follows: photosynthetic photon flux, *Φ*_*γ*_ = 225 ± 25 μmol_*γ*_ m^−2^ s^−1^; atmospheric concentration of carbon dioxide, *c*_CO2_ = 525 ± 125 ppm; air temperature, *T* = 22 ± 2 °C; relative humidity, *h*_R_ = 50 ± 10%; NSS pH = 6.0 ± 1.0. The photoperiod, *H*, was 16 h d^−1^. Further details of the growth environment are presented in Tables S1 and Figs. S3–S5. These values were selected based on specifications^11^ and growth system operation and were assumed not growth limiting by way of factors such as gas exchange and water transpiration. Harvests were performed at 11, 20, 25, 30, 35, 40 and 43 d_AE_. The plants were in the process of bolting at 43 d_AE_; as such, their data were not used. The planting density, *σ*_*N*_, was normalized to 19.2 m^−2^, and the fresh weight water fraction was assumed to be 0.95, both per their NASA reference values^11^.

Each shelf was filled to capacity with rockwool (128 pieces), with a single seed sown in each. Nutrients were added to the reservoirs at 75% of total concentration on the day of sowing to minimize the risk of non-solubilized salts and of low DO levels that had been observed in the system 3–4 days after adding the nutrients. In general, lettuce seeds do not need nutrients to germinate, which can be expected to occur within 3–10 d. At 11 d_AE_, the 55 largest and visually healthiest plants in each N condition were transplanted from the rockwool and the remaining 25% of nutrients were added to each reservoir (Tables S2 and S3). The non-transplanted seedlings from the deficient, normal, and excess N conditions weighed a total of 0.42 g, 0.42 g, and 0.38 g respectively, below the 20 g minimum required for the nitrogen assay. Harvested whole plants were weighed then frozen to − 80 °C, followed by measurement of the nitrogen content by the Dumas method^72,73^, performed commercially by Medallion Labs (Minneapolis, MN, US).

#### Nitrogen supply strategy

The decision to set a constant initial nitrate (${{{\rm{NO}}}_{3}}^{-}$) concentration while varying initial ammonium (${{{\rm{NH}}}_{4}}^{+}$) concentration in the nutrient support solution across conditions was based on several considerations. Nitrate and ammonium are the primary nitrogen forms plants uptake, with nitrate being more readily assimilated and less physiologically stressful at high concentrations. Previous studies have shown distinct plant growth responses to these forms due to their different metabolic pathways, with high ammonium levels often causing growth inhibition and stress. By setting initial nitrate constant, we ensured stable nitrogen availability, allowing us to observe the specific effects of varying ammonium levels. The experimental design aimed to mimic potential space agriculture scenarios with limited or variable nitrogen sources, enabling us to identify optimal conditions for nitrogen use efficiency.

### Parameter fitting

For each condition (normal, deficient, excess), experimental measurements of biomass and plant nitrogen content were made, and nitrogen productivity was calculated from them. The variables were experimentally measured at several instances. Let the *i*-th time instance in which a noisy measurement of the variables ${y}_{\exp ,i}$ was made be denoted as *t*_*i*_, *i* = [1, ⋯, *n*]. Note that in general the measurements can be obtained for varying instances per variable; however, here all variables were measured for the same *t*_*i*_. The *L*_2_ norm (Euclidean norm) of the difference between the predicted output of the NP model **y**_pred,i_ and the experimental values **y**_exp,i_ can be expressed as

13 $${\left\Vert {{\bf{y}}}_{{\rm{pred}}}-{{\bf{y}}}_{\exp }\right\Vert }_{2}^{2}=\mathop{\sum }\limits_{i=1}^{n}{({y}_{{\rm{pred}},i}-{y}_{\exp ,i})}^{2}$$

Parameter fitting aims to find the set of parameters that minimizes the difference between model predictions and experimental data. To achieve this, a least-squares parameter estimation problem was solved, based on the square of the Euclidean norm^74^.

14 $$\mathop{\min }\limits_{p\subseteq P}\left\Vert {{\bf{y}}}_{{\rm{pred}}}-{{\bf{y}}}_{\exp }\right\Vert_{2}^{2}$$

where $P\subset {{\mathbb{R}}}^{{n}_{p}}$ is the set of bounds for the parameter values, with *n*_*p*_ being the number of uncertain parameters. The minimization problem was solved using the SciPy package in Python, specifically with a global optimization algorithm called differential evolution^75^. This is a derivative-free method that belongs in the class of evolutionary algorithms. Once a solution is found by the algorithm, the result is further refined following a few gradient-based optimization steps using the L-BFGS method^76^.

### Sensitivity analysis

Given a multivariate ${\mathscr{P}}$ distribution for the uncertain parameters *p*, sensitivity analysis (SA) is a technique used to evaluate the degree to which the output of a model (or system) varies in response to variations of the input parameters, when the latter are drawn from the aforementioned distribution. SA is concerned with analyzing the statistical properties of the mapping *p* → *Q*(*p*), where *Q*(⋅) is some quantity of interest. In the absence of prior information about the statistical properties of the parameters, it was assumed that they followed a uniform distribution, *i.e*. ${\mathscr{P}}={\mathtt{Uniform}}(l,u)$, where *l* is a vector with the lowest values (lower bounds) that parameters *p* can attain, while *u* is the corresponding vector of upper bounds. There are two main categories of SA: (i) local sensitivity analysis (LSA) that pertains to small perturbations of the uncertain parameters and hence the corresponding “local" effect on the quantity of interest, and (ii) global sensitivity analysis (GSA) that evaluates the sensitivity of some quantity of interest over the entire distribution of the uncertain parameters. The focus was on the latter as it gave a more complete picture on the effect of uncertainty to the variation of the model predictions. More specifically, the analysis relied upon Borgonovo indices^77^, denoted by ${\mathscr{S}}$, which are based on the full distribution of some quantity of interest as opposed to their statistical moments (as done with Sobol^78^ indices). GSA methods generally collect samples in the form ${\mathscr{D}}=\{{p}_{1},Q({p}_{1}),\ldots ,{p}_{n},Q({p}_{n})\}$ and perform a series of computations to yield a set of indices for each parameter which reflect how strongly they affect the QoI. As stated earlier, here the QoI *Q* is the integral of the biomass curve over time, hence a scalar value.

GSA generally requires a large number of samples to produce reliable results. Typically, QoIs reflect physical quantities and, hence, are associated with physical constraints. Uncertainties in model parameters can lead to non-physical predictions, such as negative biomass concentrations, resulting in negative QoI integral values. Those samples should be excluded from the sensitivity analysis. A data-driven model to classify parameter combinations for physically meaningful predictions was used to accelerate the analysis; this model not only offers insight into the correlation of these parameters (*i.e*. emerging parameter patterns that lead to non-negative predictions), but also reinforces the sampling efficiency for the GSA. Although GSA typically requires between ${\mathscr{O}}(1{0}^{3})$ and ${\mathscr{O}}(1{0}^{4})$ samples for yielding reliable results^79^, the high computational cost of obtaining these samples is a concern. The approach used aimed to generate fewer samples, on the order of ${\mathscr{O}}(1{0}^{2})$, followed by the use of a classifier trained on these samples to identify trajectories likely to produce physically relevant outcomes; in this case, the classifier was a neural network^80^. The procedure is as follows:

1. Draw *N*_0_ samples from the parameters distribution ${\mathtt{Uniform}}(l,u),[{p}_{1},\cdots \,,{p}_{{N}_{0}}]$, and simulate the system to obtain the corresponding biomass curve integral values $[{Q}_{1},\cdots \,,{Q}_{{N}_{0}}]$. Only ${N}_{0}^{+}\le {N}_{0}$ samples yield positive integrals.
2. Train a classifier ${\mathscr{C}}(p)\to [0,1]$ based on all of these data; the classifier returns the probability that a given parameter combination results in a physically relevant trajectory.
3. Generate N_*_ combinations of parameters, with N_*_ ≫ *N*_0_, and pass them to the classifier. Keep all samples for which the prediction of the classifier is higher than some threshold value (probability), typically ${\mathscr{C}}({p}_{i})\ge 0.5$. The number of kept samples is ${N}_{* }^{+}\le {N}_{* }$.
4. For those kept samples, run the NP model and obtain new data $[{Q}_{1},\cdots \,,{Q}_{{N}_{* }^{+}}]$. It is expected that the vast majority of the NP model simulations yield positive integrals.
5. Finally, collect all samples that result in a positive integral from step 4, as well as the initial ${N}_{0}^{+}$ samples along with their responses, and perform the Borgonovo GSA.

## Supplementary information

Supplemental Material

## Data Availability

All data can be freely accessed from the Github repository at https://github.com/kmyates262/nitrogen-productivityand from https://zenodo.org/doi/10.5281/zenodo.10719547.

## Code availability

All code can be freely accessed from the Github repository at https://github.com/kmyates262/nitrogen-productivityand from https://zenodo.org/doi/10.5281/zenodo.10719547.
