# Supplementary material for: Nitrogen accountancy in space agriculture
Source: NPJ Microgravity. 2024 Sep 28;10:90. doi: 10.1038/s41526-024-00428-x (PMC11439006; doi:10.1038/s41526-024-00428-x)
Supplement: Supplementary file 1 — Supplemental Material [file 41526_2024_428_MOESM1_ESM.pdf]

1 Modeling

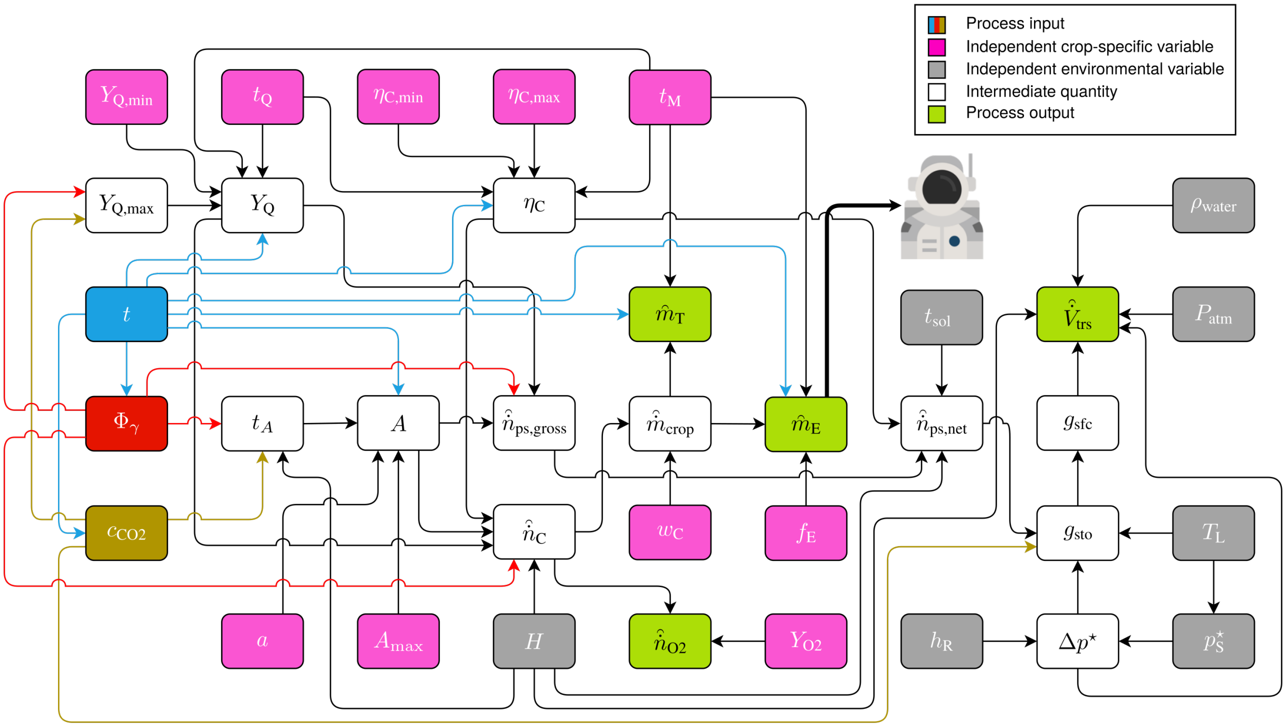

Supplementary Figure 1. Graphical breakdown of and interaction between variables for *L. sativa* MEC model. See the original source[1] and the Methods section for nomenclature. Parameters considered constant or intrinsic (e.g. molar mass of carbon, canopy type) are omitted.

2 Experimental

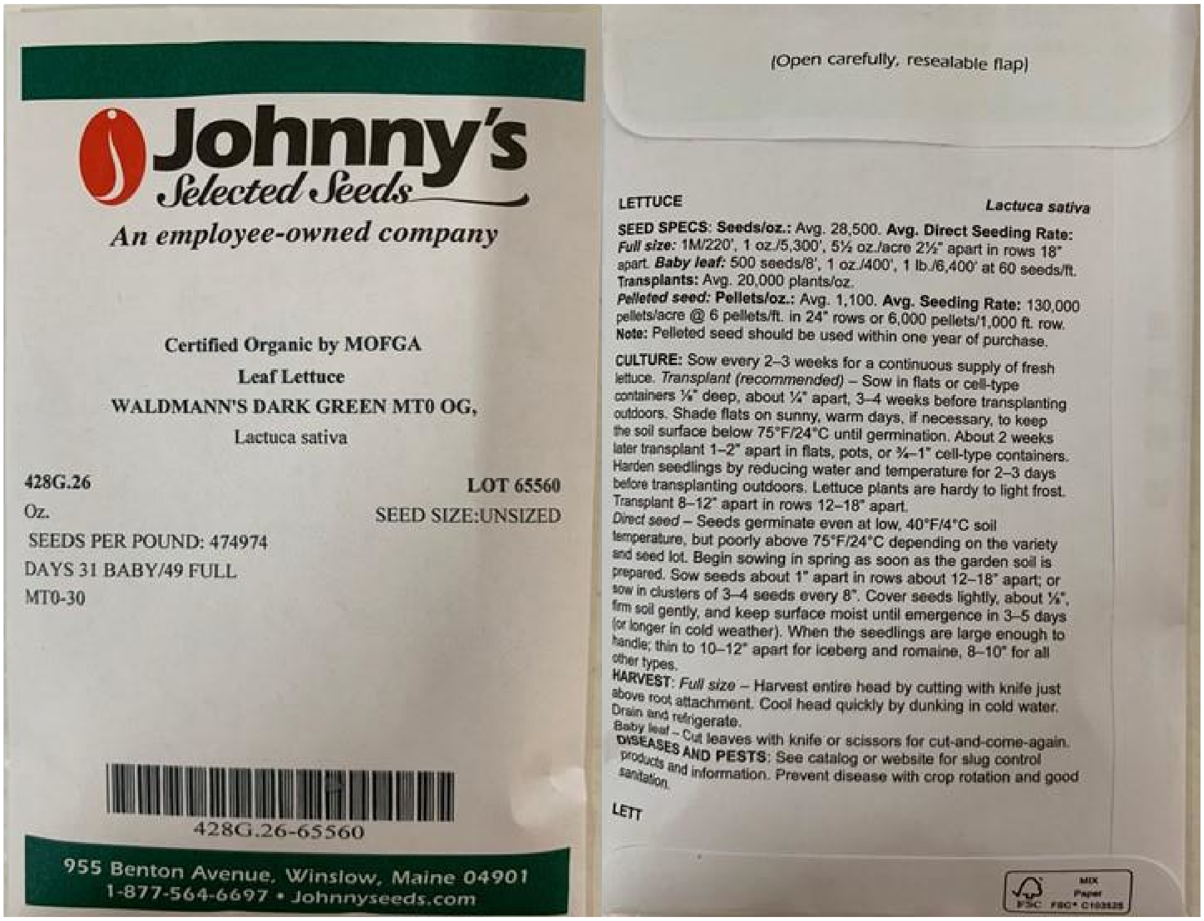

Supplementary Figure 2. Packaged seeds for *Lactuca sativa* cv. "Waldmann's Dark Green" from Johnnys Selected Seeds, product ID 428G.26, lot 66560. OG: USDA Certified Organic. MT0-30: The seed has been tested for the presence of Lettuce Mosaic Virus and none was found in a sample of at least 30,000 seeds.

Supplementary Table 1. Target and Range of Controlled Parameters

Parameter	Target	Lower Level	Upper Level
Photosynthetic photon flux [$\mu mol_\gamma m^{-2} s^{-1}$]	225	200	250
Atmospheric CO_2 concentration [ppm]	525	400	650
Air temperature [°C]	22	24	19
Humidity [%]	50	40	60
pH	6.0	5.0	7.0

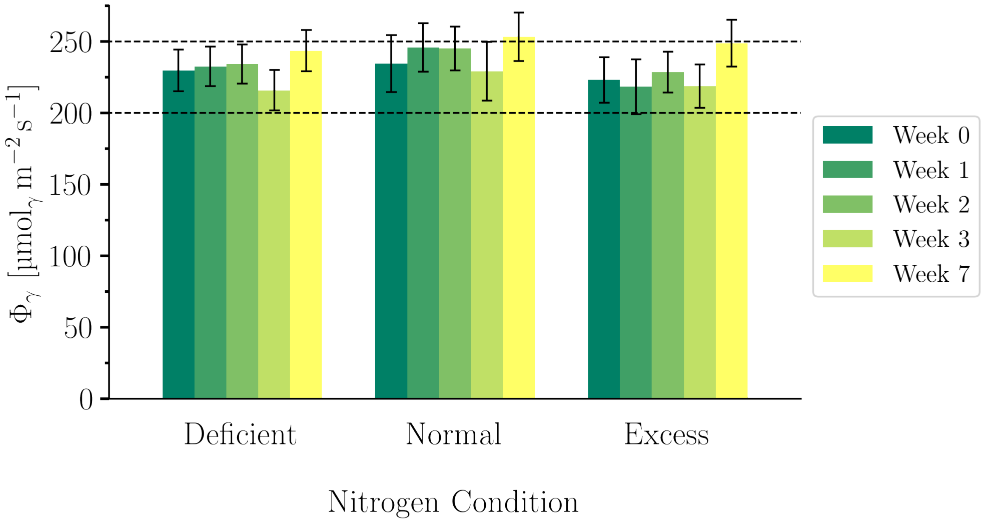

Supplementary Figure 3. Photosynthetic photon flux measurement by N condition and by week. Bars represent mean and error bars represent 1 SD. $N = 16$ for each bar. Dashed lines indicate the limits of acceptance established in the research plan.

Supplementary Table 2. Macronutrient Concentrations in NSS

Macronutrient	Derived From	Concentration [mM]		
		**Deficient**	**Normal**	**Excess**
Nitrogen (N)	Ammonium	1.5	6.5	11.5
Nitrogen (N)	Nitrate	1.0	1.0	1.0
Nitrogen (N)	Total nitrogen	2.5	7.5	12.5
Phosphorous (P)	Phosphate	1.5	1.5	1.5
Potassium (K)	Potassium salts	3.0	3.0	3.0
Magnesium (Mg)	Magnesium salts	1.5	1.5	1.5
Sulfur (S)	Combined sulfur	2.0	2.0	2.0
Calcium (Ca)	Calcium carbonate	3.0	3.0	3.0
Citrate	Citrate salts	0.4	1.8	3.0
Carbonate	Carbonate salts	2.2	2.5	3.5
Chloride	Chloride salts	0.4	2.0	2.4

Supplementary Table 3. Micronutrient Concentrations in NSS

Micronutrient	Derived From	Concentration* [µM]
Boron (B)	Boric Acid	12.9
Copper (Cu)	Copper EDTA	0.7
Iron (Fe)	Iron DTPA, Iron EDTA, Iron EDDHA	15.0
Manganese (Mn)	Manganese EDTA	2.5
Molybdenum (Mo)	Ammonium molybdate	0.3
Zinc (Zn)	Zinc EDTA	0.6

* Equal concentration in all reservoirs

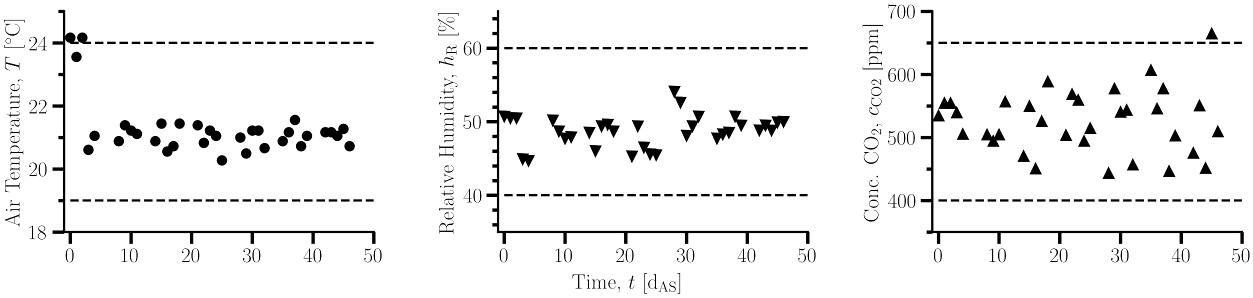

Supplementary Figure 4. Daily values of cleanroom air temperature, relative humidity, and atmospheric concentration of CO_2. Dashed lines represent the control limits established in the research plan. The first three days had higher temperatures due to a deliberate adjustment made in the system. CO_2 concentration was in the range of 400-650 ppm until the day before the final harvest when 664 ppm was measured; levels returned to normal on the next day.

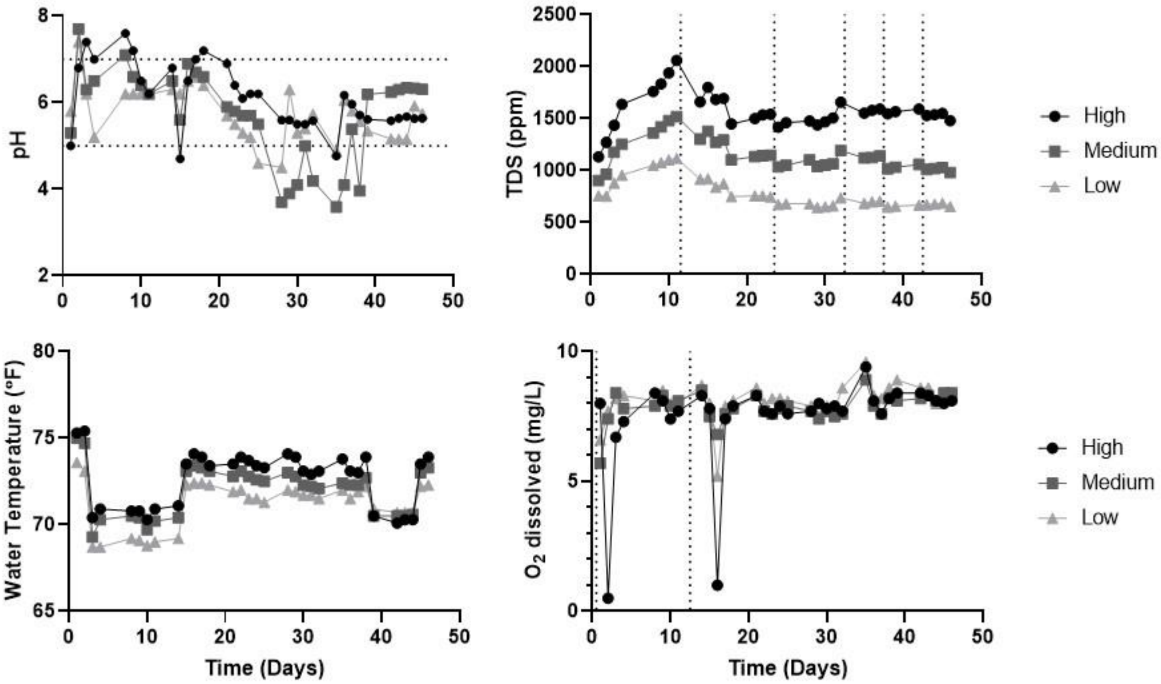

Supplementary Figure 5. Values of the NSS parameters measured manually during technicians' working hours. Horizontal dashed lines indicate the pH control limits established in the research plan. Vertical dashed lines indicate the addition of reverse osmosis (RO) water to the NSS in the total dissolved solids (TDS) graph and the addition of nutrients in the O_2 Dissolved graph. Controlling the pH proved challenging, especially for the high/excess condition, as the usual pH adjustment methods and low volumes had a small impact. In addition to this, the time for equilibrium was 24 h due to the low volumetric flow rate of water. For these reasons, some data points were out of the limits of control. The TDS showed distinct profiles dependent on the N level. The increase in TDS measurements, especially through the first four days, were due to the solubilization process (high/excess and medium/normal N levels showed intense precipitation during this period) and water evaporation. Adding RO water decreased the TDS values as expected. Variations in water temperature can be explained as follows: a) first three days: cleanroom temperature (see Figure 4); b) other fluctuations: variation in the schedule of lights with shift in the dark hours (photoperiod remaining at 16 h d^{-1}). The NSS was continuously aerated and the results were stable (93 % of the measurements are above 7.0 mg L^{-1} O_2 dissolved). Two low values were observed in the NSS for the high/excess N level and are likely due to the complete solubilization of carbonate salts (Ca and Mg carbonate) that happened 2-3 d after adding the nutrients.

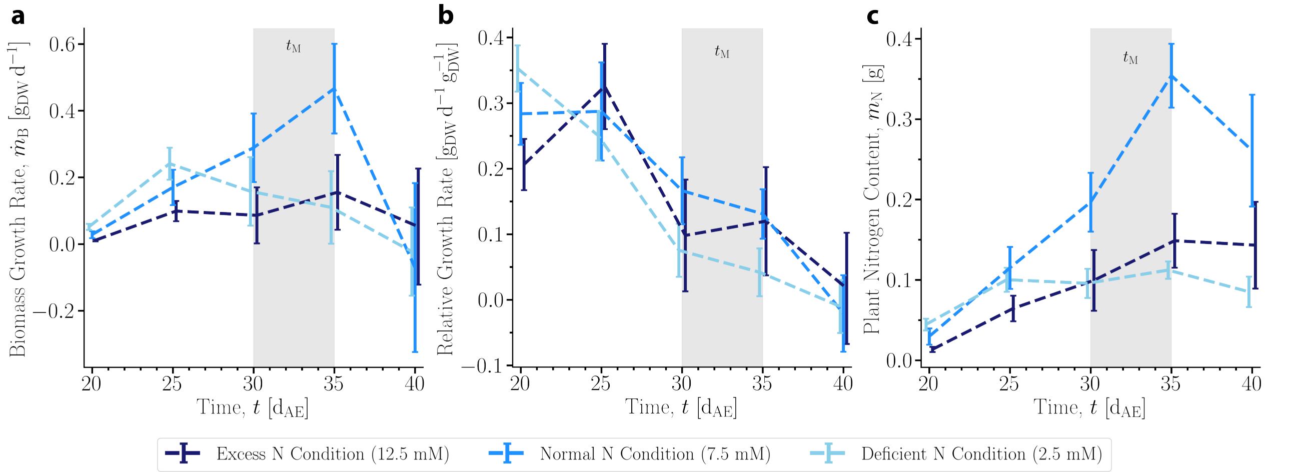

Supplementary Figure 6. (**a**) Average biomass growth rate of lettuce plants. (**b**) Average relative growth rate of lettuce plants. (**c**) Average total mass of nitrogen in lettuce plants. (**a,b,c**) For each data point, $5 \leq N \leq 10$. Error bars represent 1 SD. Area highlighted in grey is harvest time range.

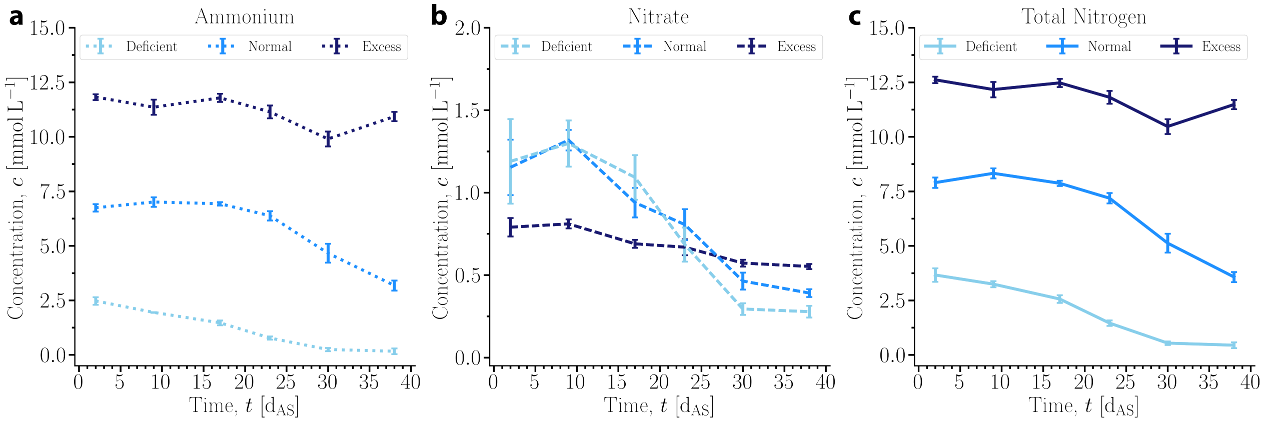

Supplementary Figure 7. (**a,b,c**) Concentrations of nitrogen measured in the NSS over time by form (ammonium, nitrate, total). Error bars represent 1 SD. For each data point, $N = 3$.

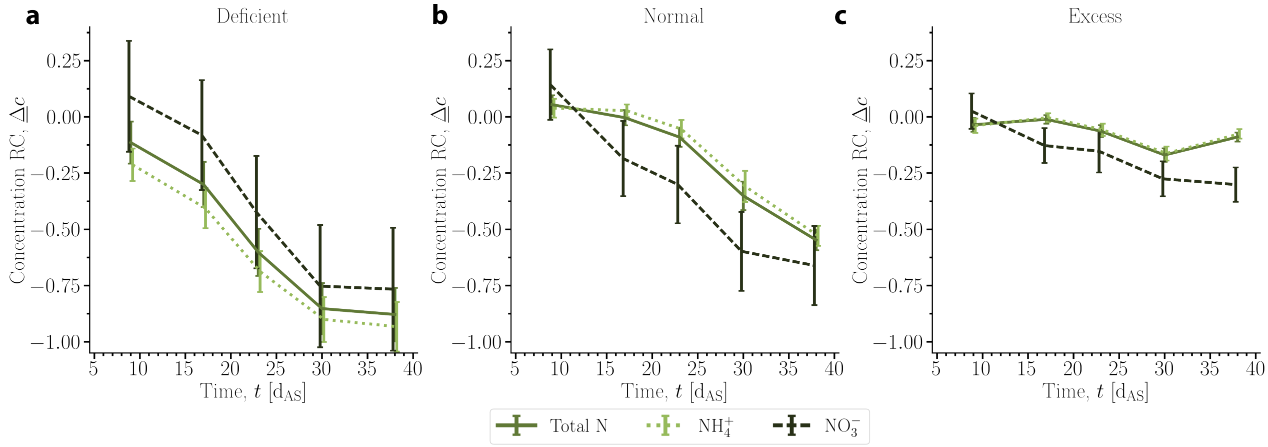

Supplementary Figure 8. (**a,b,c**) Relative change (RC) over time, denoted $\underline{\Delta}c$, where $\underline{\Delta}c \equiv [c(t) - c(t_0)]/c(t_0)$, of measured molar concentration, $c(t)$, of ammonium, nitrate, and their sum, from initially charged concentration, $c(t_0)$, in NSS for each N condition. Error bars represent one SD. $N = 3$ for each data point. Table 4 shows key quantities.

Supplementary Table 4. Times after sowing at which NH_4^+ and NO_3^- in NSS for each N condition reached half of their initial concentrations (interpolated, ±1 SD) and the overall relative changes in concentrations, $\underline{\Delta}c$, ±1 SD.

Nitrogen Condition	Nitrogen Form	Time of $\frac{1}{2}c(t_0)$ [d_{AS}]	Overall $\underline{\Delta}c$
Deficient	NH_4^+	19.0 ± 1.4	-0.93 ± 0.11
Deficient	NO_3^-	24.6 ± 4.3	-0.77 ± 0.27
Normal	NH_4^+	37.0 ± 1.5	-0.70 ± 0.04
Normal	NO_3^-	27.7 ± 3.1	-0.65 ± 0.18
Excess	NH_4^+	-	-0.16 ± 0.03
Excess	NO_3^-	-	-0.30 ± 0.08

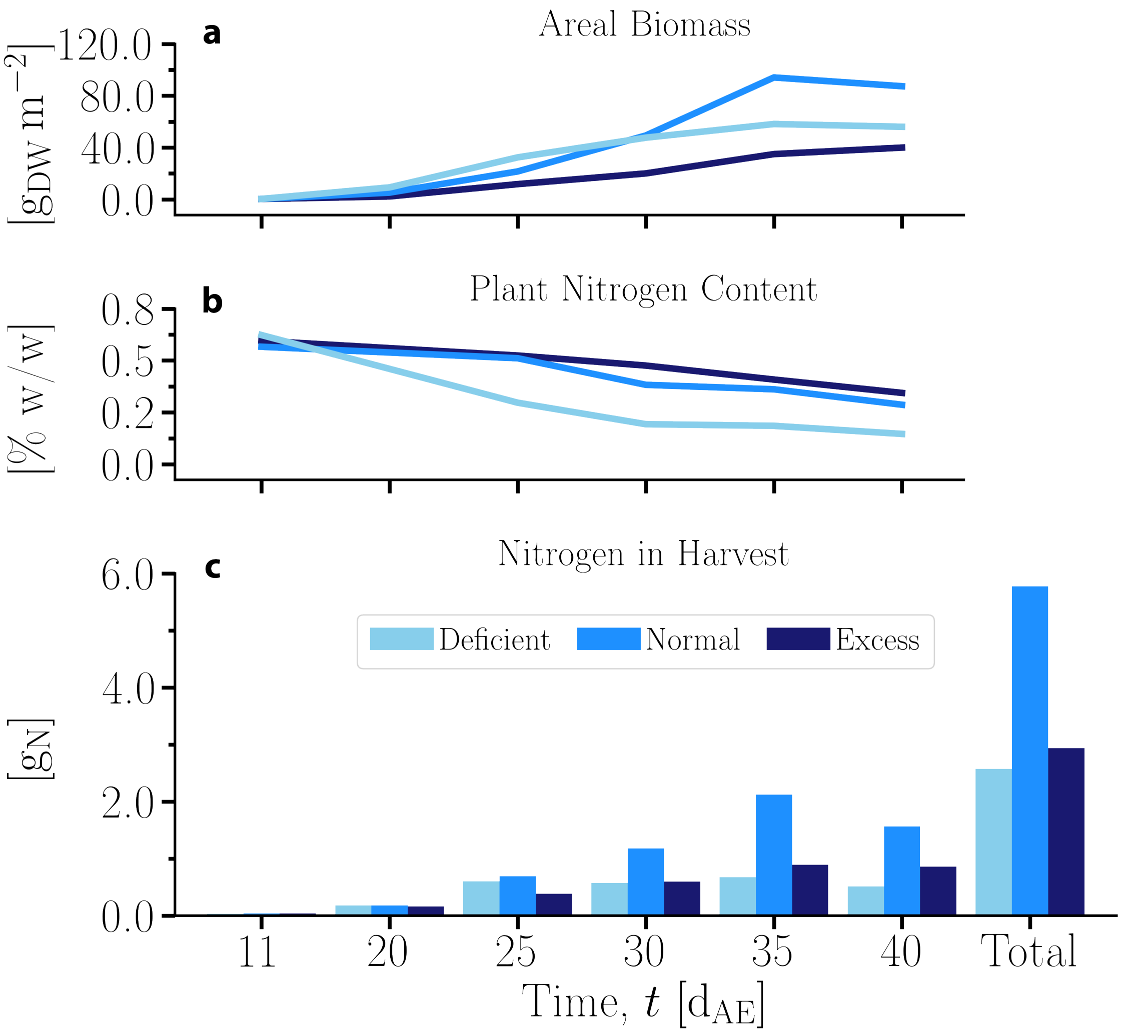

Supplementary Figure 9. (**a,b**) Biomass and nitrogen content reproduced from Figure 4 in the main text. (**c**) Total mass of nitrogen in all plants removed on harvest days for each N condition. Standard deviations were negligible. (**b, c**) Mass fraction of N in plants harvested on day 11 was extrapolated.

References

1. Ewert, M. K. & Keener, J. F. Life support baseline values and assumptions document. Tech. Rep., NASA, Washington DC (2022).
